# A practical comparison of two K-Means clustering algorithms

**DOI:** 10.1186/1471-2105-9-S6-S19

**Published:** 2008-05-28

**Authors:** Gregory A Wilkin, Xiuzhen Huang

**Affiliations:** 1 601 North 12th Street, Paragould, Arkansas 72450, USA; 2 Department of Computer Science, Arkansas State University, State University, Arkansas 72467, USA

## Abstract

**Background:**

Data clustering is a powerful technique for identifying data with similar characteristics, such as genes with similar expression patterns. However, not all implementations of clustering algorithms yield the same performance or the same clusters.

**Results:**

In this paper, we study two implementations of a general method for data clustering: *k*-means clustering. Our experimentation compares the running times and distance efficiency of Lloyd's *K*-means Clustering and the Progressive Greedy *K*-means Clustering.

**Conclusion:**

Based on our implementation, not just in processing time, but also in terms of mean squared-difference (MSD), Lloyd's *K*-means Clustering algorithm is more efficient. This analysis was performed using both a gene expression level sample and on randomly-generated datasets in three-dimensional space. However, other circumstances may dictate a different choice in some situations.

## Background

Researchers are inundated with data with little obvious information readily accessible; this is especially true in the many disciplines of the life sciences. These data may be very confusing and perplexing to biologists when viewed as a whole. To make these data more meaningful and to derive important biological understanding from these data, researchers have access to many different data processing techniques. One popular and meaningful approach is to cluster data into groups, where each group aggregates data with similar biological characteristics.

Data clustering is a very powerful technique in many application areas. Not only may the clusters have meaning themselves, but clustering allows for efficient data management techniques in that data that is grouped in the same manner will usually be accessed together. Access to data within a cluster may predict that other data in that cluster will be accessed soon; this can lead to optimized storage strategies which perform much better than if the data were randomly stored.

An easy abstraction for clustering data is based on multi-dimensional proximity relationships. While there may be other relationships among the data items, we focus on a distance relationship between data so that a meaningful and simple analytical conclusion can be made from simpler comparisons. Using proximity relationships, data is clustered in such a way that the squared-error distortion is minimized both globally and locally. The effectiveness of the algorithms analyzed are measured against this criterion. The mean squared-error distortion is defined as

*d*(*V*, *X*) = (*d*(*v*_1_, *X*)^2 ^+ *d*(*v*_2_, *X*)^2 ^+ ... + *d*(*v*_*i*_, *X*)^2 ^+ ... + *d*(*v*_*n*_, *X*)^2^)/*n*

where *X *= {*x*_1_, *x*_2_,..., *x*_*k*_} is the closest cluster center to a point in V = {*v*_1_, *v*_2_,..., *v*_*n*_} and *n *is the total number of points [1].

There are various algorithms that exist to implement clustering in terms of proximity measures. Depending on the quality of the cluster, the implementation speed of these algorithms can vary. In this article, we focus on two widely used *k*-means clustering algorithms. A *k*-means clustering algorithm can be formally defined as a function that receives as input a set of points in multi-dimensional space and a number, *k*, of desired centers or cluster representatives; one area of active research is the issue of optimally "seeding" the algorithm with the proper value of *k *and the starting locations of the *k *cluster centers. With this input, the algorithm produces an output set of point sets such that each point set has a defined center that minimizes the cumulative distance to the center of all points in that set, for all the possible choices of each set.

We have implemented two versions of the *k*-means clustering algorithm: Lloyd's *K*-means Clustering and Progressive Greedy *K*-means Clustering. The former is a relatively faster algorithm and is fairly straightforward. The latter is a more conservative approach and can run for a much longer time but can sometimes yield better results in terms of distance measures.

We first describe these algorithms, then we examine these algorithms and discuss some experimental results. These results are analyzed based on the running time for the algorithms and the mean squared-error distortion and are compared in terms of complexity and efficiency.

## Methods

### Algorithm description: Lloyd's *K*-means Clustering algorithm

Lloyd's *K*-means Clustering algorithm was designed by S. P. Lloyd [2]. Given a number *k*, separate all data in a given partition into *k *separate clusters, each with a center that acts as a representative. There are iterations that reset these centers then reassign each point to the closest center. Then the next iteration repeats until the centers do not move. The algorithm is as follows [1]:

*1. Assign each data point to the cluster C_i _corresponding to the closest cluster representative x_i_(1 ≤ i ≤ k*)

*2. After the assignments of all n data points, compute new cluster representatives according to the center of gravity of each cluster*.

While the Lloyd's algorithm often converges to a local minimum of the squared error distortion rather than the global minimum [1], it is the faster of the two algorithms discussed in this paper.

We used **C **as the programming language to implement this algorithm using two primary structures for the points: an array of points that is dynamically declared when the user specifies the input points and arrays for each of *k *centers. These latter arrays for each center themselves have arrays within them – one for each dimensional in a multi-dimensional space – for the points that are assigned to that particular center (for our analysis, we have used three-dimensional points).

### Algorithm description: Progressive Greedy *K*-means Clustering algorithm

The Progressive Greedy *K*-means Clustering algorithm is similar to Lloyd's in that it searches for the best center of gravity for each point, but it assigns points to a center based on a different technique. In each iteration, Lloyd's algorithm reassigns a point to a new center and then readjusts the centers accordingly. The Progressive Greedy approach does not act upon every point in each iteration; rather the point which would most benefit moving to another cluster is reassigned. Every iteration in the Progressive Greedy algorithm calculates the "cost" of every point in terms of a Euclidean distance (in three-dimensional space), i.e.,

√[(x_1 _- x_2_)^2 ^+ (y_1 _- y_2_)^2 ^+ (z_1 _- z_2_)^2^]

Each point *p *= (*x*_*p*_, *y*_*p*_, *z*_*p*_) has a cost associated with it in terms of the current center *C*_*i *_= (*x*_*i*_, *y*_*i*_, *z*_*i*_) to which it belongs. The point is a candidate to be moved if the Eculidean distance cost can be reduced by moving that point from one cluster *C*_*i *_to another cluster *C*_*j *_= (*x*_*j*_, *y*_*j*_, *z*_*j*_) with that cluster having a closer center. In other words, a point is a candidate to be moved from *C*_*i *_to *C*_*j *_if

√[(x_i _- x_p_)^2 ^+ (y_i _- y_p_)^2 ^+ (z_i _- z_p_)^2^] - √[(x_j _- x_p_)^2 ^+ (y_j _- y_p_)^2 ^+ (z_j _- z_p_)^2^]

is greater than 0. Once all the candidates are calculated, the point with the largest difference is then moved. If no point has a difference value greater than 0, the algorithm is finished.

Each iteration in the Progressive Greedy *K*-means Clustering algorithm does the following:

*1. Calculate the cost of moving each point to each of the other cluster centers as well as the cost of its current cluster center. For every point, store the best change if less than the cost of its current cluster center*.

*2. If there is a point with a best change, move it. If there is more than one, pick the one point that when moved sees the greatest improvement*.

*3. If nothing else can be done, finished*.

The Progressive Greedy *K*-means Clustering is slower, but the sacrifice is an attempt to minimize the squared-error distortion mentioned earlier.

The implementation of Progressive *K*-means clustering uses the same C data structures as was used for Lloyd's.

## Results

### Analysis of biological data

M. B. Eisen, *et. al. *[3] were one of the first groups to apply the clustering approach to the analysis the gene expression data.

We applied both clustering algorithms to the analysis of microarray data. The clustering algorithms classified gene expression data into clusters such that functionally-related genes are grouped together. In the following example [1], the expression information of ten genes is recorded at three different times (see Table 1). The distance matrix of the ten genes was calculated based on the Euclidean distance in three-dimensional space. The clustering algorithms grouped the gene expression data into clusters satisfying the following two conditions [1]:

**Table 1:** Expression levels of ten genes at three different times.

**Gene**	**1 hr**	**2 hr**	**3 hr**
**g**_1_	10.0	8.0	10.0
**g**_2_	10.0	0.0	9.0
**g**_3_	4.0	8.5	3.0
**g**_4_	9.5	0.5	8.5
**g**_5_	4.5	8.5	2.5
**g**_6_	10.5	9.0	12.0
**g**_7_	5.0	8.5	11.0
**g**_8_	2.7	8.7	2.0
**g**_9_	9.7	2.0	9.0
**g**_10_	10.2	1.0	9.2

• within a cluster, any two genes should be highly similar to each other (i.e., the distance between them should be small; this condition is called *homogeneity*), and

• any two genes from different clusters should be very different from each other (i.e., the distance between them should be large; this condition is called *separation*).

Both algorithms yielded the same three clusters of the ten genes as follows: {g_1_, g_6_, g_7_}, {g_3_, g_5_, g_8_}, and {g_2_, g_4_, g_9_, g_10_}. Tables 2 and 3, respectively, are the running time comparisons and mean squared-distance comparisons of the two clustering algorithms applied to these biological data.

**Table 2:** Running time comparison in seconds for different *k *values.

	**k = 2**	**k = 3**	**k = 4**	**k = 5**
**Lloyd's**	0.465	0.470	0.480	0.620
**Progressive**	0.140	0.207	0.250	0.280

**Table 3:** MSD comparisons for different *k *values (actual values).

	**k = 2**	**k = 3**	**k = 4**	**k = 5**
Lloyd min MSD	0.75	0.69	0.00	0.69
**Lloyd global avg. MSD**	**9.94**	**2.81**	**1.95**	**2.81**
Lloyd max MSD	19.13	7.00	7.00	7.00
Progressive min MSD	0.75	0.75	0.13	0.69
**Progressive global avg. MSD**	**9.94**	**5.13**	**2.69**	**3.81**
Progressive max MSD	19.13	7.64	7.00	9.98

### Analysis of a randomly-generated data set

**Figure 1:**
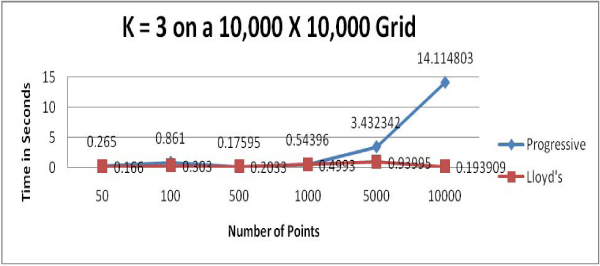
Running time comparison when *k *= 3.

**Figure 2:**
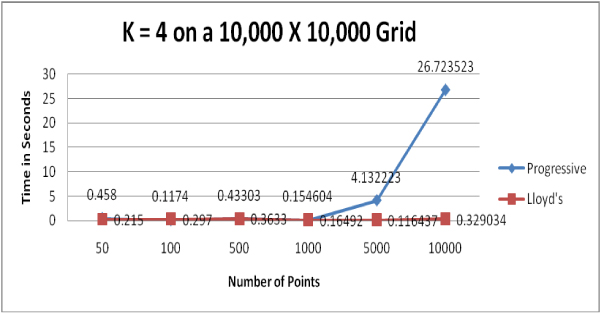
Running time comparison when *k *= 4.

**Figure 3:**
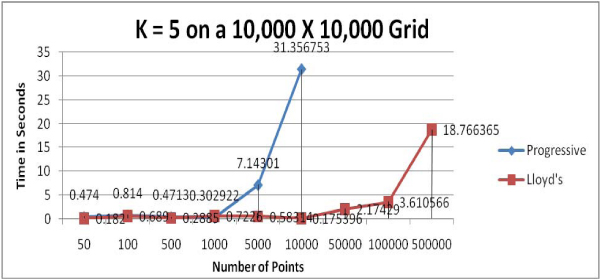
Running time comparison when *k *= 5 (excludes the running times of Progressive Greedy algorithm when the number of points exceeds 10,000).

**Figure 4:**
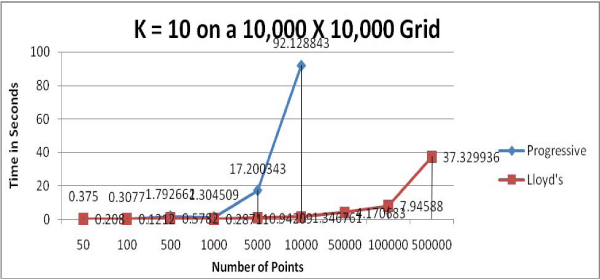
Running time comparison when *k *= 10 (excludes the running times of Progressive Greedy algorithm when the number of points exceeds 10,000).

We used computer-generated random points to test the two clustering algorithms; presumably, this data represents few natural clusters which should present close to a "worst case" for the clustering algorithms. Figures 1 to 4 show the running time comparisons of various runs using different values of *k *and different numbers of points. Each individual value in these Figures is a mean time of multiple runs and is expressed in terms of seconds, though what is important here is the relative size of these values.

A comparison of mean square differences are shown in Tables 4 and 5 using different numbers of points and *k *values of 5 and 10, respectively. In these Tables, the maximum and minimum local cluster mean squares are shown alongside the general global average MSD.

**Table 4:** MSD comparisons with different number of points when *k *= 5 (in millions of actual values).

**for *k *= 5**	**50 pts**	**100 pts**	**1000 pts**	**5000 pts**	**10000 pts**
Lloyd min MSD	4.078	5.222	8.116	7.997	8.089
**Lloyd global avg. MSD**	**7.142**	**7.423**	**9.604**	**9.915**	**9.804**
Lloyd max MSD	9.622	9.250	11.439	11.259	10.875
Progressive min MSD	7.070	5.750	7.539	8.031	8.071
**Progressive global avg. MSD**	**8.610**	**8.395**	**9.715**	**9.916**	**9.804**
Progressive max MSD	10.442	10.240	11.761	11.247	10.859

**Table 5:** MSD comparisons with different number of points when *k *= 10 (in millions of actual values).

**for *k *= 10**	**50 pts**	**100 pts**	**1000 pts**	**5000 pts**	**10000 pts**
Lloyd min MSD	0.745	2.633	4.905	4.823	5.015
**Lloyd global avg. MSD**	**4.355**	**4.845**	**5.519**	**5.538**	**5.588**
Lloyd max MSD	7.350	6.916	6.497	5.983	6.097
Progressive min MSD	0.745	2.633	4.578	4.602	4.713
**Progressive global avg. MSD**	**5.266**	**4.978**	**5.564**	**5.503**	**5.547**
Progressive max MSD	9.721	6.970	6.222	6.106	6.149

## Conclusion

The advantage of Lloyd's *K*-means Clustering algorithm compared to the Progressive Greedy *K*-means Clustering algorithm is clear from the above comparisons. Based on our implementation, not just in processing time, but also in terms of mean squared-difference, Lloyd's *K*-means Clustering algorithm is more efficient. For very large data sets, Lloyd's algorithm definitely works faster. When the number of points exceeds 10000, the Progressive Greedy *K*-means Clustering algorithm needs optimization to even to be able to handle the very large floating point values associated with finding the mean squared-difference. Without optimization, Progressive Greedy *K*-means Clustering would not even run without generating floating point exception errors. We therefore conclude that Lloyd's *K*-means Clustering algorithm seems to be the better algorithm. However, other circumstances may dictate a different choice in some situations.

## Competing interests

The authors declare that they have no competing interests.

## Authors' contributions

GAW carried out the *k*-means clustering algorithm design and implementation. XH participated in the design and applications of the algorithms. Both authors have read and approved the final manuscript.
